# Epsin1 modulates synaptic vesicle retrieval capacity at CNS synapses

**DOI:** 10.1038/srep31997

**Published:** 2016-08-25

**Authors:** Jae Won Kyung, Jae Ryul Bae, Dae-Hwan Kim, Woo Keun Song, Sung Hyun Kim

**Affiliations:** 1 Department of Biomedical Science, Graduate School, Kyung Hee University, Seoul 02447, South Korea; 2 Institute of Pharmaceutical Science and Technology, Hanyang University, Ansan, 15588 South Korea; 3 School of Life Science, Bio Imaging and Cell Dynamics Research Center, Gwangju Institute of Science and Technology, Gwangju 61005 South Korea; 4 Department of Physiology, Kyung Hee University, School of Medicine, Seoul 02447, South Korea; 5 Neurodegeneration Control Research Center, Seoul, 02447, South Korea

## Abstract

Synaptic vesicle retrieval is an essential process for continuous maintenance of neural information flow after synaptic transmission. Epsin1, originally identified as an EPS15-interacting protein, is a major component of clathrin-mediated endocytosis. However, the role of Epsin1 in synaptic vesicle endocytosis at CNS synapses remains elusive. Here, we showed significantly altered synaptic vesicle endocytosis in neurons transfected with shRNA targeting Epsin1 during/after neural activity. Endocytosis was effectively restored by introducing shRNA-insensitive Epsin1 into Epsin1-depleted neurons. Domain studies performed on neurons in which domain deletion mutants of Epsin1 were introduced after Epsin1 knockdown revealed that ENTH, CLAP, and NPFs are essential for synaptic vesicle endocytosis, whereas UIMs are not. Strikingly, the efficacy of the rate of synaptic vesicle retrieval (the “endocytic capacity”) was significantly decreased in the absence of Epsin1. Thus, Epsin1 is required for proper synaptic vesicle retrieval and modulates the endocytic capacity of synaptic vesicles.

Neuronal information flow via neurotransmission is sustained by continuous supply of synaptic vesicles. Several pathways for synaptic vesicle retrieval have been identified, including kiss-and-run^1^, bulk endocytosis^2^, ultrafast endocytosis^3^, and clathrin-mediated endocytosis^4^. Each vesicle retrieval pathway is activated under normal or specific conditions in nerve terminals. Among these, clathrin-mediated endocytosis of synaptic vesicles is one of the most efficient retrieval pathways, with significant evidence showing that molecular players in this endocytic pathway are required for proper synaptic vesicle endocytosis^4^,^5^,^6^,^7^,^8^. Epsin was initially identified as a binding partner of epidermal growth factor receptor substrate 15 (EPS15), one of the components of clathrin-mediated endocytosis^9^. Three Epsin isoforms have been identified (Epsin1, Epsin2, and Epsin3) in mammals to date, among which Epsin1 is highly enriched and represents the dominant isoform in brain^9^. Epsin1 is composed of four conserved domains, which are all involved in the course of endocytosis through interactions with a series of binding partners in various cells. For example, the Epsin N-Terminal Homology (ENTH) domain of Epsin1 binds membrane content, such as PI(4,5)P_2_^10^. Ubiquitin-Interacting Motifs (UIM) are responsible for specific cargo selection and contain the monoubiquitination sorting signal of endocytosis^11^,^12^. The clathrin/AP2 binding (CLAP) domain associates with endocytic core machineries, clathrin and AP-2 (Adaptor Protein-2 complex), and the asparagine-proline-phenylalanine (NPF) motif at the C-terminus interacts with EPS15 homology (EH) domain-containing proteins, such as EPS15 and intersectin^9^. Epsin is highly conserved among various species. Membrane binding via the ENTH domain of Epsin (Ent1, Ent2) in yeast is essential for endocytosis^13^, and this domain generates membrane curvature^14^,^15^. Ubiquitin-dependent endocytosis by Epsin1 (liquid facets) in *Drosophila* is required for synaptic growth^16^. Moreover, this region is specifically required for virus internalization^17^. Epsin1 (Epn-1) in *C. elegans* is involved in regulation of receptor signaling and receptor internalization^18^,^19^. ENTH and CLAP inhibition via injection of domain-specific antibodies in lamprey reticulospinal synapse led to a decreased number of synaptic vesicles, implying that synaptic vesicle endocytosis is impaired by blocking Epsin function^20^. Accumulating reports on orthologs of Epsin in various species strongly indicate that Epsin1 mainly participates in endocytosis. However, the potential function of Epsin1 in synaptic vesicle endocytosis at CNS synapses remains to be established.

In the present study, we investigated whether Epsin1 plays a role in synaptic vesicle endocytosis using shRNA-mediated ablation of Epsin1 along with pHluorin-conjugated synaptic vesicle proteins in primary cultured rat hippocampal neurons and a high-fidelity optical imaging system. Our experiments clearly demonstrated that upon depletion of Epsin1, the kinetics of synaptic vesicle endocytosis is severely impaired. Following Epsin1 depletion in neurons, replacement with deletion mutants of individual ENTH, CLAP, and NPFs domains, but not UIMs, of Epsin1 led to post-stimulus synaptic vesicle retrieval. Intriguingly, endocytic capacity induced by various neural activities was significantly altered in Epsin1 knockdown (Epsin1 KD) neurons.

## Results

### Epsin1 accumulates significantly at nerve terminals

**Figure 1:**
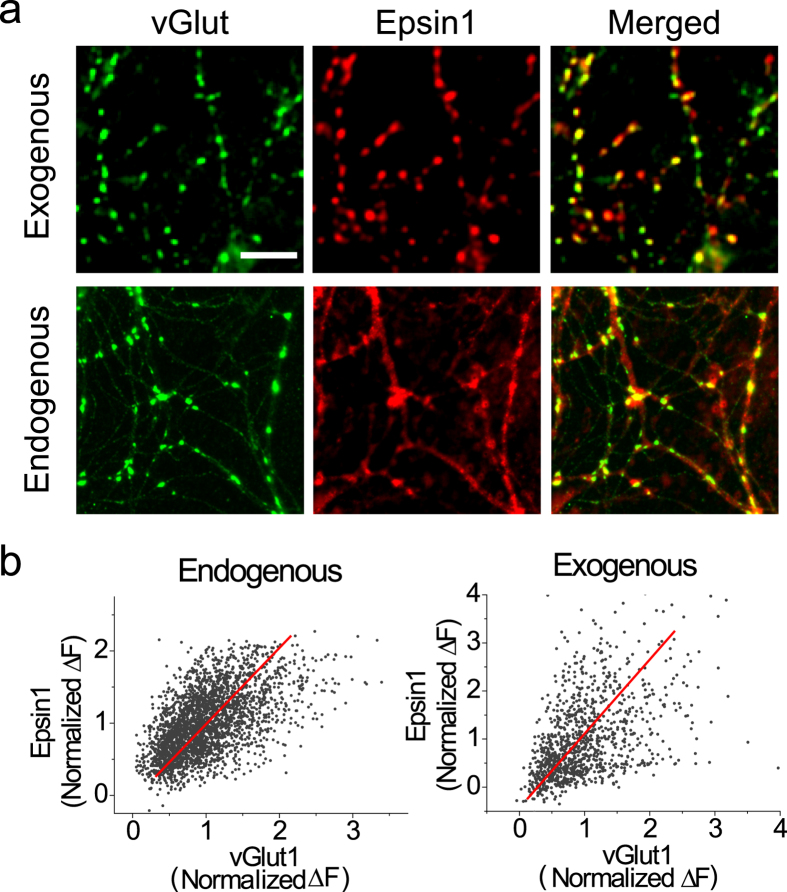
Epsin1 is highly enriched at nerve terminals. (**a**) Representative images of exogenous vGlut1 (vG-pH) and Epsin1 (Epsin1-mKate2) [top] or endogenous vGlut1 (green) and Epsin1 (red) [bottom] in primary cultured hippocampal neurons. Neurons were fixed at 14–18 days *in vitro* (DIV) and stained with anti-vGlut1 (green) and anti-Epsin1 (red) for endogenous vGlut1 and Epsin1, respectively. Scale bar, 5 μm. (**b**) Distribution of Epsin1 corresponds to each nerve terminal in terms of endogenous (left) and exogenous (right) levels. Higher levels of Epsin1 localize at synapse as a function of synaptic size.

Epsin1, originally identified as a binding partner of EPS15, is highly expressed in the brain^9^. Since Epsin1 is a known adaptor protein in clathrin-mediated endocytosis (CME), we examined whether the protein is enriched at presynaptic terminals in primary cultured hippocampal neurons with high levels of CME. We exogenously co-expressed mKate2-conjugated Epsin1 with vGlut1-pHluorin, a presynaptic protein, in primary cultured hippocampal neurons. Neurons at 14 days *in vitro* (DIV) were fixed and visualized via microscopy. Epsin1-mKate2 co-localized significantly with vGlut1-pHluorin as punctate patterns, indicating localization of Epsin1 at nerve terminals (Fig. 1a, top). To exclude the possibility that the observed distribution of Epsin1-mKate2 is an overexpression artifact, we additionally performed immunohistochemical analysis of endogenous Epsin1 distribution. As shown in Fig. 1a (bottom), endogenous Epsin1 was highly co-localized with endogenous vGlut1. Moreover, this distribution was positively correlated with the intensity of vGlut1, a presynaptic marker (Fig. 1b). The data show that Epsin1 is enriched at nerve terminals, supporting its requirement for presynaptic functions.

### Surface accumulation of vGlut1-pHluorin is increased while synaptic vesicle pool size is decreased in Epsin1-depleted neurons

**Figure 2:**
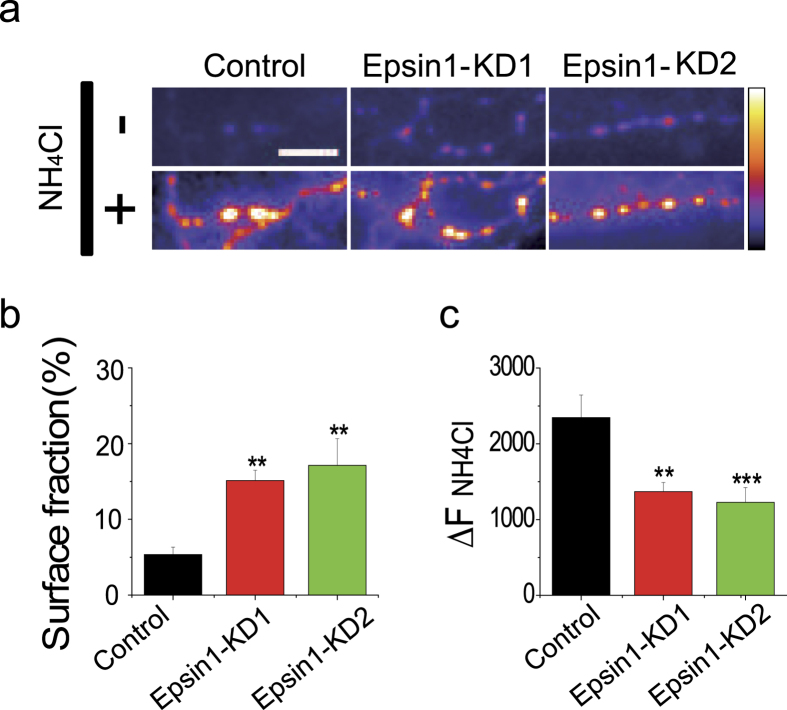
Ablation of Epsin1 induces an increase in the surface fraction of vGlut1-pHluorin and decrease in total synaptic vesicle pool size. (**a**) Representative snapshots of vGlut1-pHluorin at the resting state and after applying NH_4_Cl in control and Epsin1-KD1 or -KD2 neurons, (top) NH_4_Cl−, absence of NH_4_Cl indicates steady-state under resting conditions and (bottom) NH_4_Cl+, presence of NH_4_Cl indicates application of NH_4_Cl to reveal synaptic bouton with the total vesicle population. Scale bar, 5 μm. (**b**) Mean values of surface accumulation at rest in control, Epsin1-KD1, and Epsin1-KD2 neurons. Surface accumulation of vG-pH was measured by calculating the fold change in response to NH_4_Cl application. The surface fraction in control neurons was 5.35 ± 0.97% (n = 9), while that measured in Epsin1-KD1 and Epsin1-KD2 neurons was 15.12 ± 1.36% (n = 9) and 17.12 ± 3.53% (n = 7), respectively. (**c**) Total vesicle pool size (TVS) was determined by measuring absolute changes in fluorescence intensity in response to application of NH_4_Cl under the same imaging conditions. In Epsin1-KD1 neurons, TVS was reduced to ~58%, compared to that in control neurons. 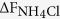 (Con) = 2346 ± 298 a.u. (n = 7), 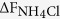 (Epsin1-KD1) = 1368 ± 120 a.u (n = 9), 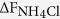 (Epsin1-KD2) = 1226 ± 194 (n = 7), **p < 0.01, ***p < 0.001. One-way ANOVA.

To determine the specific function of Epsin1 at CNS synapses, we employed the shRNA-based gene knockdown system. This method has been utilized to delineate functions of synaptic proteins at the single-neuron level^5^,^21^,^22^,^23^. Our method of gene delivery into primary culture neurons had relatively low efficiency so that we are able to trace as an expression reporter at the single-neuron level. Accordingly, we employed the pHluorin-tagged synaptic vesicle reporter in combination with shRNA targeting Epsin1. pHluorin, a mutant form of green fluorescence protein (GFP) that can be quenched by protonation, was fused to the luminal domain of the synaptic vesicle protein, vGlut1^24^. Neurons were transfected with or without shRNA-Epsin1, and experiments conducted between 6 and 10 days after transfection. Expression of Epsin1 in individual transfected neurons was measured retrospectively via quantitative analysis of immunofluorescence using the Epsin1 antibody, and intensity normalized to that of non-transfected neurons on the same slide. Epsin1 levels were successfully reduced by ~80% in both shRNA1-Epsin1 (Epsin1-KD1) and shRNA2-Epsin1 (Epsin1-KD2)-transfected neurons (Supplementary Fig. 1). Neurons expressing vGlut1-pHluorin (vG-pH) showed a very low surface fraction (~4%) at rest, since the majority of reporter protein was localized at the internal acidic vesicle pool at synapses^5^,^25^. We further investigated whether Epsin1 ablation alters the surface fraction of vG-pH under resting conditions. In control neurons transfected with vG-pH alone, surface accumulation of vG-pH was 5.35 ± 0.97%, in agreement with previous reports^5^,^25^. However in neurons co-transfected with vG-pH and shRNA-Epsin1-KD1 or -KD2, the surface fraction of vG-pH was significantly increased by ~3-fold (Epsin1-KD1 = 15.12 ± 1.36% and Epsin1-KD2 = 17.12 ± 3.53%) (Fig. 2a,b). These data consistently support previous reports that impairment of endocytosis via deficiency of endocytic proteins results in accumulation of synaptic vesicle proteins on the synaptic membrane surface^5^,^26^. Next, we examined the relative size of the total synaptic vesicle pool in the presence and absence of Epsin1. Total fluorescence changes after application of NH_4_Cl represent the size of the synaptic vesicle pool labeled with vG-pH^5^. In Epsin1-depleted neurons, the total fluorescence change (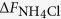) was significantly reduced to ~58% that in control neurons (Fig. 2c). Our results indicate that the synaptic vesicle pool size is decreased in Epsin1 KD neurons, as reported previously in lamprey reticulospinal synapse^20^.

### Synaptic vesicle endocytosis is severely altered during and after neural activity in Epsin1-depleted neurons

**Figure 3:**
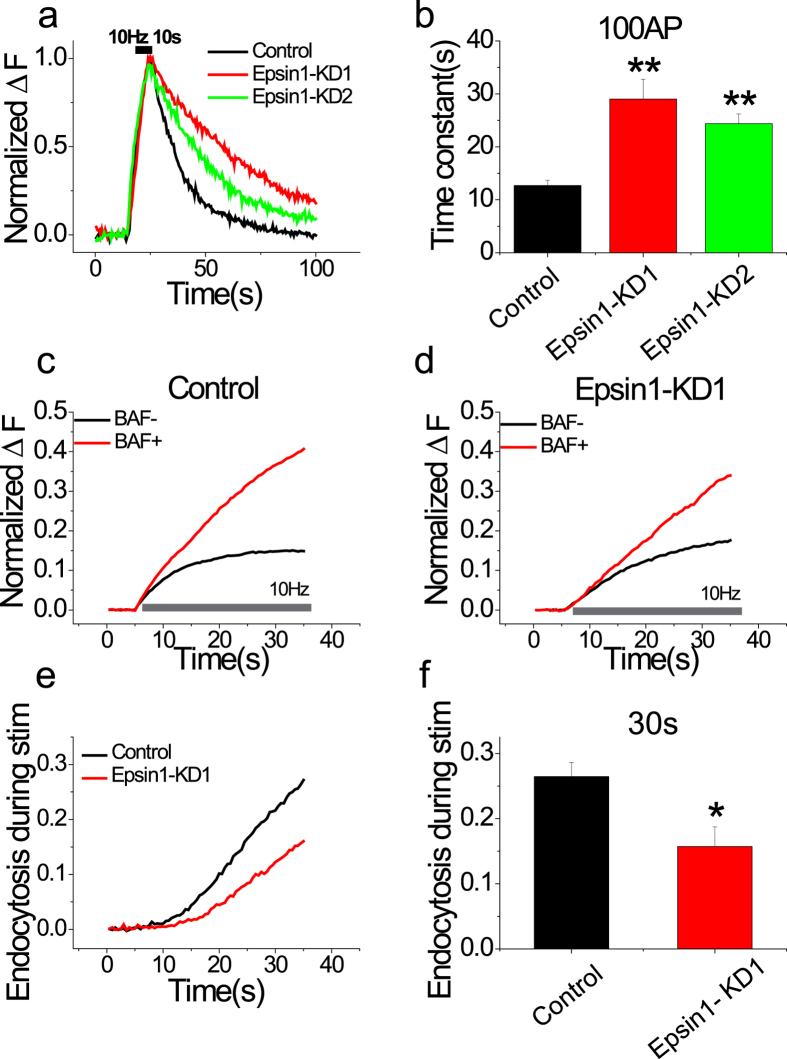
Synaptic vesicle endocytosis during and after neural activities is altered in Epsin1 KD neurons. (**a**) Representative traces of the vG-pH response to 100 action potential (AP) firing from control (black), Epsin1-KD1 (red) and Epsin1-KD2 (green) neurons. Neurons transfected with vG-pH and Epsin1-KD1 or Epsin1-KD2 were stimulated with 100 AP at 10 Hz. (**b**) Mean values of post-stimulus endocytic time constants from control, Epsin1-KD1, and Epsin1-KD2 neurons (τ_endo con_ = 12.69 ± 0.98 s, n = 9; τ_endo Epsin1-KD1_ = 29.02 ± 3.78 s, n = 9; τ_endo Epsin1-KD2_ = 24.41 ± 1.80 s, n = 9). (**c,d**) Representative traces of vG-pH response to 300 AP at 10 Hz with (red)/without (black) bafilomycin A1 (BAF) from control (**c**) and Epsin1-KD1 (**d**) neurons. (**e,f**) Endocytosis during neural activity is strongly suppressed in Epsin1-KD1 neurons. Endocytosis during stimulation in control neurons (black). Endocytosis during stimulation was analyzed as ΔF_300Baf+_ -ΔF_300Baf−_. Con _endo_ = 26.4 ± 0.2% (n = 9), Epsin1KD-1 _endo_ = 15.7 ± 0.3% (n = 9), *p < 0.05, **p < 0.01; One-way ANOVA.

To ascertain whether Epsin1 is involved in synaptic vesicle recycling at CNS synapses, we employed vGlut1-pHluorin (vG-pH) in combination with shRNA targeting Epsin1 expressed in hippocampal neurons. We used the pHluorin assay to monitor synaptic vesicle endocytosis as previously described^5^. Recycling assays conducted between 6 and 13 days after transfection revealed a strong decrease in Epsin1 expression (Supplementary Fig. 1). To examine the effects of Epsin1 knockdown on synaptic vesicle endocytosis, 100 action potentials (AP) firing at 10 Hz were delivered to initially induce synaptic vesicle exocytosis, and endocytosis monitored after application of stimuli in Epsin1-depleted neurons. The kinetics of fluorescence decay caused by endocytosis of synaptic vesicles in Epsin1-KD1 and Epsin1-KD2 neurons was ~2–3–fold slower (τ_endo Epsin1-KD1_ = 29.02 ± 3.78 s, τ_endo__Epsin1-KD2_ = 24.41 ± 1.80 s), than that of control neurons (τ_endo_ _control_ = 12.69 ± 0.98 s) (Fig. 3a,b). We additionally investigated synaptic vesicle endocytosis during neuronal activities by comparing the responses of vG-pH to 30 s stimulation at 10 Hz in the presence and absence of bafilomycin A1, a proton pump antagonist. In the absence of bafilomycin A1, a vG-pH signal indicates net surface accumulation of balance of between exocytosis and endocytosis during activities at the given time (30 s). In the presence of bafilomycin A1, the vG-pH signal indicates total exocytosis during the given activities, since reacidification of synaptic vesicles is blocked. Comparison of these two signals allowed us to measure the total amount of endocytosis during a given activity time^7^,^27^,^28^. All of these endocytosis assays were performed at 30 °C. To test whether the endocytic defect in Epsin1 KD neurons was influenced by temperature, we repeated our endocytosis assays at 25 °C and 35 °C. Although the kinetics of endocytosis in control and Epsin1 KD neurons were globally affected by alteration in temperature, Epsin1 KD neurons consistently showed an endocytic defect compared to control neurons at all tested temperatures (Supplementary Fig. 2). It is note that Epsin1 knockdown did not affect the rate of synaptic vesicle exocytosis (Supplementary Fig. 3). In control neurons, the level of endocytosis after 30 s stimulation at 10 Hz was similar to ~30% total recycling vesicle pool (Fig. 3c,e,f), in agreement with previous reports^5^. However, in Epsin1-depleted neurons, endocytosis during neural activities was reduced to half that of the control group (Fig. 3d–f). Thus our data imply that Epsin1 is required for proper endocytic uptake of synaptic vesicles during and after neural activity.

### Defects in synaptic vesicle retrieval in shRNA-Epsin1-transfected neurons can be rescued by expression of shRNA-insensitive Epsin1

**Figure 4:**
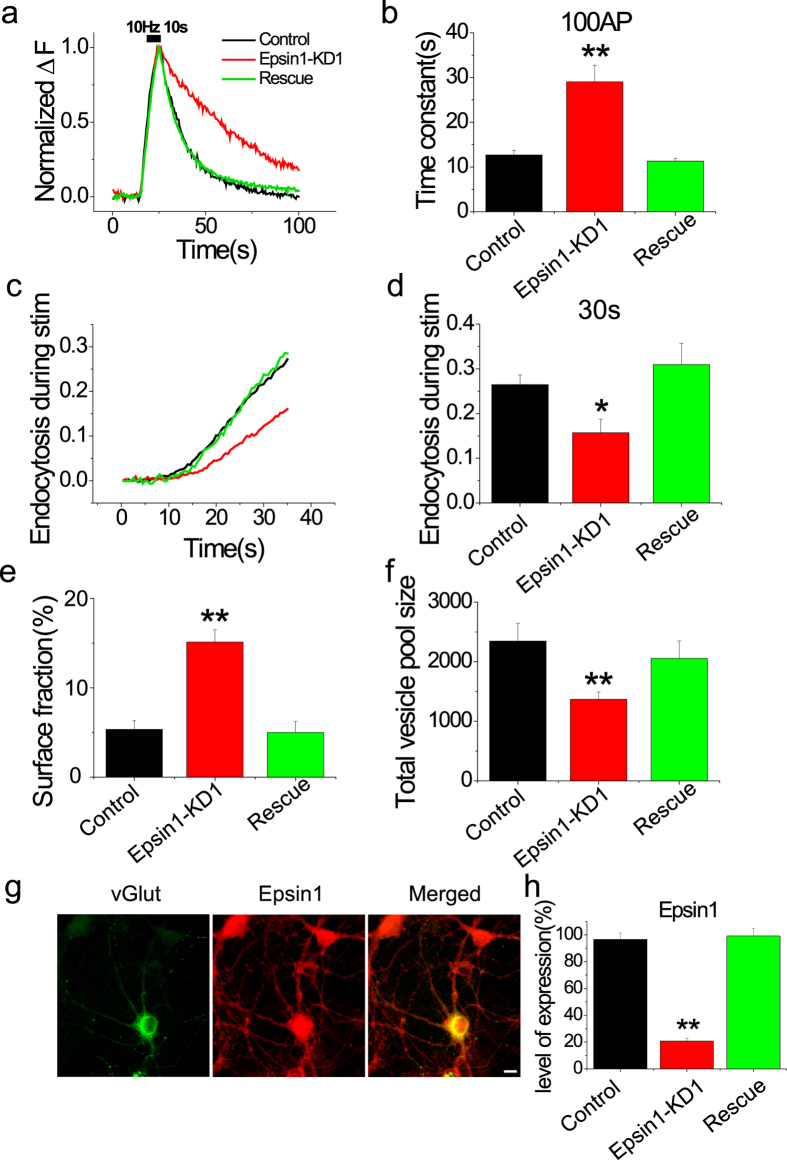
Defects in endocytic phenotype are restored by expressing shRNA-insensitive Epsin1 in Epsin1 KD neurons. (**a**) Representative vG-pH trace responses to 100 AP from control (black), Epsin1-KD1 (red), and rescue (green) neurons. For rescue experiments, neurons were transfected with shRNA1-Epsin1, shRNA1-insensitive Epsin1, and vG-pH. (**b**) Mean values of post-stimulus endocytic time constants from control, Epsin1-KD1, and rescue neurons. (**c**) Endocytosis during stimulation is restored upon shRNA-insensitive Epsin1 expression in Epsin1-KD1 neurons, calculated as for Fig. 3. (d) The amount of endocytosis during stimulation (22.83 ± 2.9%) is similar to that of the control. (**e,f**) Surface accumulation of vG-pH and total vesicle pool size (TVS) are restored to control levels in rescue neurons. Surface fraction _rescue_ 5.0 ± 1.2% (n = 7), TVS _rescue_ = 2056 ± 292 a.u (n = 7). (**g**) Representative images of rescue neurons. Neurons expressing shRNA1-Epsin1, shRNA-insensitive Epsin1 and vG-pH were fixed and stained with anti-Epsin1 (red). (**h**) Expression of Epsin1 is almost completely restored (99.2 ± 5.7%) to levels in non-transfected neurons by re-expressing shRNA-insensitive Epsin1 in Epsin1-KD1 neurons. *p < 0.05, **p < 0.01. One-way ANOVA; ns means non-significant.

To ascertain whether endocytic defects result specifically from loss of Epsin1 and not an off-target effect of shRNA, we introduced shRNA-insensitive Epsin1 in Epsin1-ablated neurons. Notably, the kinetics of post-stimulus endocytosis and amount of endocytosis during stimulation were almost restored to control cell levels under these conditions (Fig. 4a–d). In addition, surface accumulation of vG-pH and total vesicle pool size were rescued in shRNA-insensitive Epsin1-expressing neurons (Fig. 4e,f). Consistently, functional rescue was effectively recapitulated with recovery of Epsin1 expression (Fig. 4g,h). Our results clearly indicate that the defects in synaptic vesicle endocytosis induced by shRNA targeting Epsin1 result from depletion of Epsin1 expression.

### ENTH, CLAP and NPFs domains are responsible for synaptic vesicle retrieval, but not the UIMs domain

**Figure 5:**
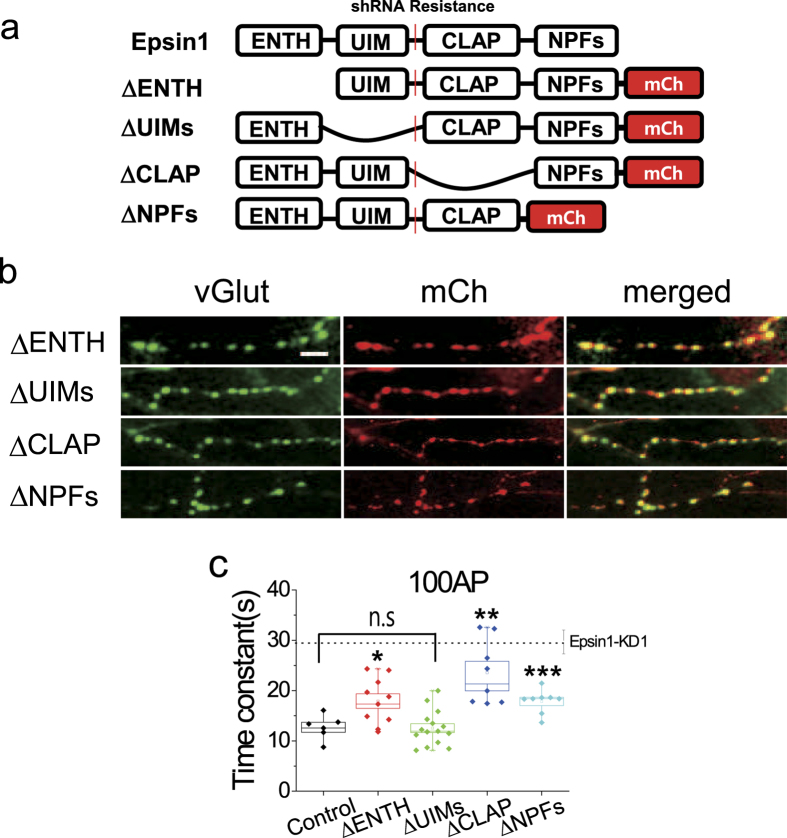
ENTH, CLAP, and NPFs, but not UIMs of Epsin1 are essential for synaptic vesicle endocytosis. (**a**) Schematic diagram of each domain deletion mutant of Epsin1 (ΔENTH, ΔUIMs, ΔCLAP and ΔNPFs). (**b**) All deletion mutants are located placed at nerve termini of cultured hippocampal neurons. Neurons co-transfected with each deletion mutant, vG-pH and shRNA1-Epsin1 were fixed after 14~21 DIV. Scale bar, 5 μm. (**c**) Mean values of endocytic time constants from control, ΔENTH, ΔUIM, ΔCLAP, and ΔNPFs rescue neurons. In ΔENTH, ΔCLAP and ΔNPFs rescue neurons, kinetics of endocytosis is impaired, but not in ΔUIMs rescue cells. τ_endo con_ = 12.69 ± 0.99 s, n = 9; τ_endo ΔENTH_ = 17.90 ± 1.44 s, n = 10; τ_endo ΔUIMs_ = 12.53 ± 0.88 s, n = 15; τ_endo ΔCLAP_ = 23.57 ± 2.25 s, n = 8; τ_endo_ _ΔNPFs_ = 17.85 ± 0.82 s, n = 8. *p < 0.05, **p < 0.01, ***p < 0.001. One-way ANOVA; ns means non-significant.

Epsin1 is composed of four distinct domains that specifically interact with different binding partners. The ENTH domain is specific for PI(4,5)P_2_ and actin cytoskeleton^29^, UIM for ubiquitin, CLAP for clathrin and AP-2, and NPFs motif for EH domain-containing protein (i.e., EPS15). To determine the specific roles of individual domains of Epsin1 in synaptic vesicle endocytosis, we generated corresponding domain deletion mutants with a shRNA-insensitive Epsin1 backbone (Epsin1 ΔENTH, ΔUIMs, ΔCLAP, and ΔNPFs) (Fig. 5a). Neurons were simultaneously transfected with vG-pH, Epsin1-KD1, and individual domain deletion mutants to replace endogenous Epsin1. Firstly, we confirmed that each deletion mutant is targeted at the nerve terminal (Fig. 5b). Next, the synaptic vesicle recycling assay was performed. In Epsin1 ΔENTH-expressing and Epsin1 ΔNPFs-expressing neurons, the post-stimulus endocytic time constant was significantly slower, compared to that of control neurons (τ_endo Control_ = 12.69 ± 0.99 s, τ_endo Epsin1 ΔENTH_ = 17.90 ± 1.44 s, τ_endo Epsin1ΔNPFs_ = 17.85 ± 0.82 s). In Epsin1 ΔCLAP-expressing neurons, the time constant of endocytosis was even more severely impaired (τ_endo Epsin1ΔCLAP_ = 23.57 ± 2.25 s). Interestingly, however, in Epsin1 ΔUIMs-expressing neurons, no endocytic defects were evident (τ_endo Epsin1ΔUIMs_ = 12.53 ± 0.88 s) (Fig. 5c). Accordingly, we propose that ENTH, CLAP, and NPFs play important roles in post-stimulus endocytosis of synaptic vesicles, but not the UIMs domain.

### Endocytic capacity is altered in Epsin1 KD neurons

**Figure 6:**
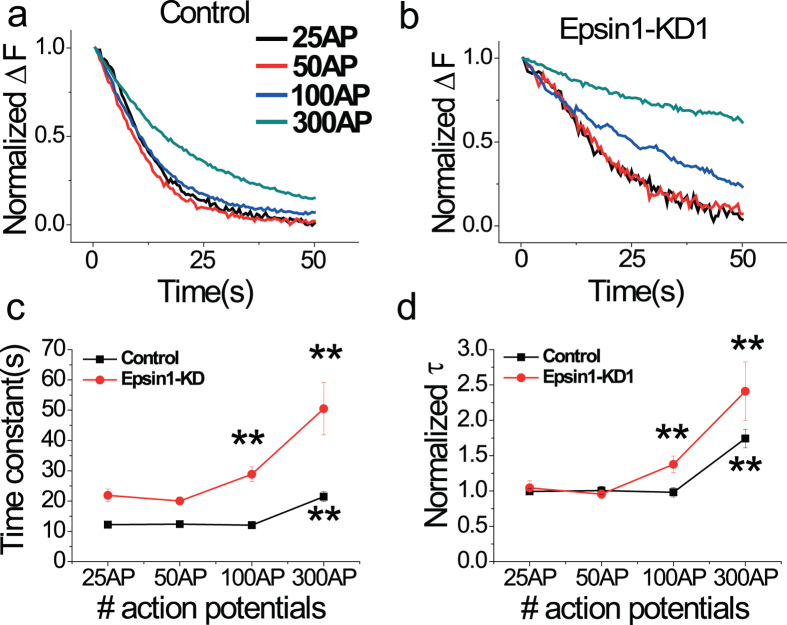
Endocytic capacity is decreased in Epsin1 KD neurons. (**a,b**) Representative post-stimulus traces of vG-pH response to 25 (black), 50 (red), 100 (blue), and 300 (green) AP, respectively, from control (**a**) and Epsin1-KD1 (**b**) neurons. The kinetics of post-stimulus endocytosis was measured in neurons subjected to a range of stimuli (25, 50, 100, and 300 AP). (**c**) Mean values of endocytic time constants at 25, 50, 100 and 300 AP in control and Epsin1-KD1 neurons. The time constant in Epsin1-KD1 neurons is altered from 100AP but in control neurons from 300 AP. τ_endo con 25AP_ = 12.23 ± 0.53 s, τ_endo con 50AP_ = 12.38 ± 0.71 s, τ_endo con 100AP_ = 12.07 ± 0.85 s, τ_endo con 300AP_ = 21.44 ± 1.63 s, n = 8 ; τ_endo Epsin1-KD1 25AP_ = 21.87 ± 2.09 s, τ_endo Epsin1-KD1 50AP_ = 20.03 ± 1.29 s, τ_endo Epsin1-KD1 100AP_ = 28.83 ± 2.45 s, τ_endo Epsin1-KD1 300AP_ = 50.50 ± 8.63 s, n = 10. (**d**) Normalized time constants in control and Epsin1-KD1 neurons under exposure to various stimuli. The time constant is normalized by the average value of 25–50 AP under each condition. Overlay of normalized time constants under various stimulation conditions in control (black) and Epsin1-KD1 (red) neurons. Endocytic capacity is significantly decreased in Epsin1-KD1 neurons. **p < 0.01, One-way ANOVA.

The kinetics of endocytosis is generally invariant regarding the number of vesicles endocytosed, which does not exceed the critical range of the total vesicle pool at synapses^30^. “Endocytic capacity” refers to the maximum point of this critical range. When the number of vesicles accumulating on the surface by activity-driven synaptic vesicle exocytosis and awaiting endocytosis exceeds this point, the kinetics of endocytosis begins to slow down^30^. However, if the number of synaptic vesicles awaiting endocytosis is below this point, it shows less variant of time constant. This capacity of endocytosis is modulated by Ca^2+^ influx, which corresponds to neural activity^30^. However, the molecular players contributing to this process are not known as yet. Lower endocytosis rates are generally thought to be due to a shortage of endocytic machinery, as endocytic demand caused by surface accumulation via a large volume of synaptic vesicle exocytosis increases above a critical point. Using this model, we examined whether Epsin1 ablation alters the endocytic capacity of synaptic vesicles. Specifically, neurons were stimulated to various extents (from 25 to 300 AP) sufficient to observe the endocytic capacity range. In control neurons, post-stimulus endocytic time constants were consistent from 25 to 100 AP at 10 Hz, and started slowing down at 300 AP, 10 Hz (Fig. 6a,c,d). However, in Epsin1-KD1 neurons, post-stimulus endocytosis was consistent only during very weak stimulation activity from 25 to 50 AP. Upon application of 100 AP, post-stimulus endocytosis was significantly slower, and consistently, endocytic time constants were severely slower at 300 AP at 10 Hz (Fig. 6b–d,). In addition, this alteration of endocytic capacity under different levels of stimulation was consistently observed at temperatures of 25 °C and 35 °C (Supplementary Fig. 5), clearly implying that Epsin1 modulates the capacity of synaptic vesicle endocytosis.

### Endocytic capacity does not depend on specific cargo proteins in Epsin1 KD neurons

**Figure 7:**
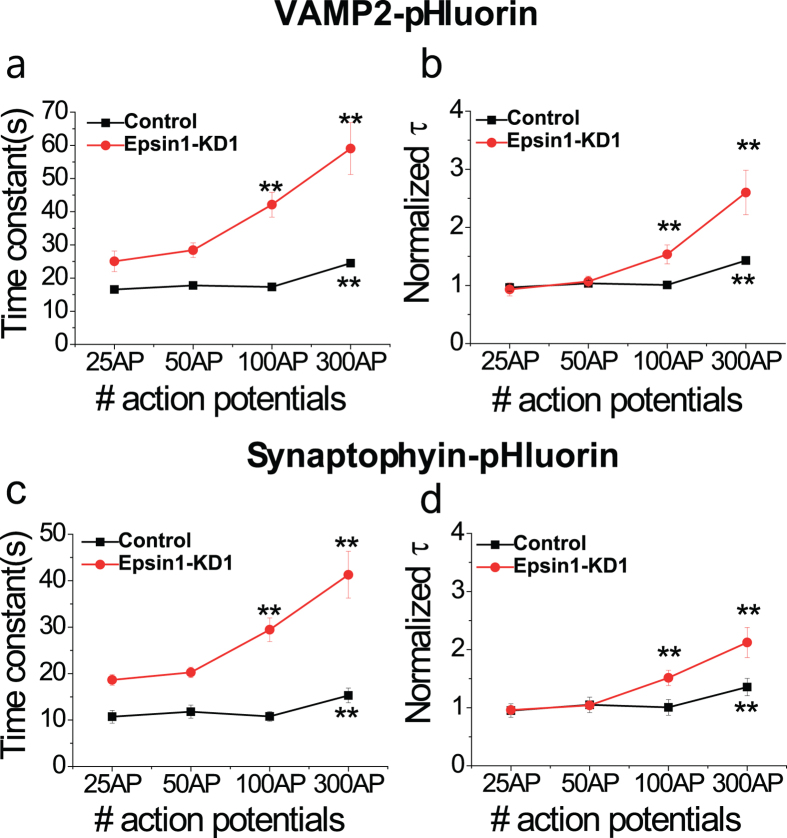
Alterations in endocytic capacity are not cargo protein-dependent in Epsin1- depleted neurons. (**a,c**) Mean values of time constants of post-stimulus endocytosis of VAMP2-pHluorin (**a**; VAMP2-pH) or synaptophysin-pHluorin (**c**; physin-pH) in response to various stimuli (25, 50, 100, and 300 AP, respectively) in control and Epsin1-KD neurons. τ_endo VAMP2-pH Con 25AP_ = 16.55 ± 1.07 s, τ_endo VAMP2-pH Con 50AP_ = 17.76 ± 0.88 s, τ_endo VAMP2-pH Con 100AP_ = 17.30 ± 0.57 s, τ_endo VAMP2-pH Con 300AP_ = 24.49 ± 1.08 s, n = 6 ; τ_endo VAMP2-pH-Epsin1-KD1 25AP_ = 25.05 ± 3.10 s, τ_endo VAMP2-pH-Epsin1-KD1 50AP_ = 28.39 ± 2.18 s, τ_endo VAMP2-pH-Epsin1-KD1 100AP_ = 42.13 ± 3.72 s, τ_endo VAMP2-pH Epsin1-KD1 300AP_ = 59.02 ± 7.80 s, n = 9. τ_endo physin-pH-con 25AP_ = 10.71 ± 1.36 s, τ_endo physin -pH-con 50AP_ = 11.80 ± 1.42 s, τ_endo physin -pH-con 100AP_ = 10.77 ± 1.03 s, τ_endo physin -pH-con 300AP_ = 15.29 ± 1.57 s, n = 6 ; τ_endo physin-pH-Epsin1-KD1 25AP_ = 18.64 ± 1.14 s, τ_endo physin-pH-Epsin1-KD1 50AP_ = 20.26 ± 1.08 s, τ_endo physin-pH-Epsin1-KD1 100AP_ = 29.43 ± 2.54 s, τ_endo physin-pH-Epsin1-KD1 300AP_ = 41.28 ± 5.037 s, n = 8. **p < 0.01. One-way ANOVA. (**b,d**) Normalized mean values of endocytic time constants of VAMP2-pH or physin-pH in control and Epsin1-KD neurons. **p < 0.01, One-way ANOVA.

Synaptic Vesicles (SV) contain about 10 different types of SV major cargo proteins^31^. After fusion of SV to the target region of the plasma membrane (i.e., active zone), the cargo content of SV has to be maintained by endocytic retrieval. Experiments were performed to determine whether changes in endocytic capacity in Epsin1-KD1 neurons are dependent on specific cargo proteins. To this end, we employed two other pHluorin systems that conjugate with two major SV cargo proteins, VAMP2 (VAMP2-pH) and synaptophysin (physin-pH). Neurons transfected with VAMP2-pH or physin-pH with/without Epsin1-KD1 were monitored for post-stimulus endocytosis under the same scheme as vG-pH expressing neurons. As shown in Fig. 7, in cells without Epsin1, the endocytic capacities of VAMP2 and synaptophysin were decreased, while in cells with Epsin1, endocytic capacities were not different to that of control cells with vG-pH. We also observed the expression levels of SV cargo proteins in Epsin1 KD neurons. As shown in Supplementary Figure 6, expression levels of SV cargo proteins were not altered in Epsin1 KD neurons. Thus, alterations in endocytic capacity of Epsin1-depleted neurons do not appear to depend on specific cargo proteins in SV, implying that Epsin1 is a general modulator of SV endocytic capacity.

## Discussion

Since its identification as an EPS15 binding partner, Epsin has been implicated in clathrin-mediated endocytosis in various cellular systems, including cancer cells^32^,^33^,^34^ and developmental processes^35^,^36^. In this study, we investigated the function of Epsin1 in synaptic vesicle endocytosis (SVE) at CNS synapses by employing a high-fidelity optical imaging system in combination with the pHluorin assay. Three intriguing phenotypes of synaptic vesicle endocytosis at nerve terminals were observed in the absence of Epsin1.

Our first observation was that in neurons depleted of Epsin1, a known endocytic component, the kinetics of post-stimulus endocytosis was severely altered under various temperature conditions. In general, when neurons are depleted via RNA interference (knockdown) or gene knockout of several known endocytic proteins, such as AP-2, endophilin and synaptojanin, the kinetics of synaptic vesicle endocytosis is impaired^7^,^8^,^37^. In addition to post-stimulus defects, severe endocytic defects during neural activity were evident. Our data collectively indicate that Epsin1 is critical for proper synaptic vesicle endocytosis during and after neural activity.

Next, domain studies were performed to determine the specific domain(s) essential for synaptic vesicle endocytosis. As shown in Fig. 5, ENTH, CLAP, and NPFs domains contributed to post-stimulus synaptic vesicle endocytosis, but not the UIMs motif. ENTH is known to generate membrane curvature, and ENTH of Ent1 and Ent2 in yeast is essential for endocytosis^13^. The CLAP domain is a key region that interacts with core endocytic machinery (Clathrin and AP-2). Replacement of endogenous Epsin1 with ΔENTH or ΔCLAP mutant protein after Epsin1 KD in cultured neurons led to strong impairment of the synaptic vesicle endocytosis rate. Our results are consistent with the report by Jakobsson *et al*.^20^ that functional blocking via injecting antibodies against the ENTH or CLAP domain impairs synaptic vesicle recycling. Similar results were obtained upon replacement with the ΔNPFs Epsin mutant, suggesting that Epsin1 association with scaffolding proteins, such as EPS15 and intersectin, is also required for proper synaptic vesicle endocytosis. Surprisingly, however, replacement with the ΔUIMs Epsin1 mutant in neurons induced near-normal post-stimulus synaptic vesicle endocytosis, although endocytosis during stimulation was slightly defective, suggesting that the UIMs domain of Epsin1 in hippocampal neurons contributes to synaptic vesicle endocytosis during stimulus, but not post-stimulus (supplementary Fig. 4). Mono-ubiquitination has been established as a specific cargo recognition signal for endocytosis in some cell types. UIM in Epsin1 is required for internalization of virus and other species (i.e. *Drosophila*, yeast), but not in *C. elegans.* The reason why the UIMs domain does not play a functional role in post-stimulus synaptic vesicle endocytosis is currently unclear, but may be explained by the fact that ubiquitination of synaptic proteins is regulated very rapidly (on a seconds scale) by neural activity-dependent Ca^2+^ influx^38^. Thus, the role of UIMs in synaptic vesicle endocytosis may be highly dependent on neuronal activity.

The third and most striking discovery was that Epsin1 is able to modulate the capacity of synaptic vesicle endocytosis. Synaptic vesicle endocytosis is tightly coupled with surface accumulation of synaptic vesicles via synaptic vesicle exocytosis under various neural stimuli. The rate of synaptic vesicle endocytosis (efficiency of endocytosis) is constant in a range of modest repetitive neural activities, since endocytic machinery may provide efficient recycling under these conditions. However, efficiency of endocytosis may be affected upon exceeding the range of surface accumulation of synaptic vesicles owing to prolonged neural stimulation. Endocytic capacity is also modulated by the intracellular Ca^2+^ level, although the underlying mechanism remains to be established^30^. To date, the molecular players responsible for endocytic capacity have not been identified. Importantly, our results disclosed that in the absence of Epsin1, the endocytic capacity of synaptic vesicles is altered, even within the modest range of neural activity (100 AP), while that of control neurons remains unchanged under the same conditions. Since the cytosolic Ca^2+^ level can control the endocytic capacity at nerve terminals^30^. we hypothesized that Epsin1 depletion could alter the activity-dependent Ca^2+^ influx. To test this possibility, we monitored activity-dependent Ca^2+^ influxes under various levels of stimulation (25, 50, and 100 AP) using the presynaptic terminal specific genetic Ca^2+^ indicator, synaptophysin-GCaMP6f. However, the presynaptic Ca^2+^ influx in Epsin1 KD neurons was not different from that of control neurons (Supplementary Fig. 7). Moreover, Epsin1 KD neurons do not show any change in the expression levels of SV cargo proteins (Supplementary Fig. 6), and the alteration of endocytic capacity in Epsin1 KD neurons was found to be independent of specific cargoes (Fig. 7). Collectively, these findings show that Epsin1 modulates endocytic capacity independent of the levels of Ca^2+^ and SV cargo proteins.

To sustain the efficiency of vesicle recycling at a constant speed during a specific range of modest activity, presumably, orchestration of each step of the endocytic process by interacting core and accessory endocytic proteins is required in parallel. So far, there are no reports of endocytic proteins that affect endocytic capacity, although the kinetics of synaptic vesicle endocytosis is generally slower in protein-depleted conditions^22^. Notably, our experiments revealed that Epsin1 depletion affects not only the speed of recycling but also endocytic capacity, implying that Epsin1 participates in the proper endocytic process of synaptic vesicles and additionally modulates endocytic capacity in various neural activities.

The favored model is that Epsin1 is relatively widely involved in endocytosis. The protein initially binds and generates curvature of membrane via the ENTH domain and further interacts with the core machinery of endocytosis, including clathrin and AP-2 via CLAP, as well as scaffolding proteins, such as EPS15 and intersectin, via the NPFs motif. Since replacement with a single domain mutant of Epsin1 did not affect endocytic capacity, we propose that the observed effect of Epsin1 is attributable to its broad contribution to endocytosis via multiple interactions (Supplementary Fig. 8).

Modulation of endocytic capacity may facilitate the effective maintenance of synaptic information flow via useful synaptic vesicle recycling and preparation, although the particular rate-limiting step for regeneration of ready-to-release vesicles after neural activity remains to be established. Our findings on Epsin1-dependent endocytic capacity during various neural activities may therefore aid in the development of strategies to further control maintenance of synaptic communication.

We cannot completely rule out the possibility of compensatory effects from other isoforms, Epsin2 or Epsin3, which are also expressed in brain, albeit at relatively low levels. Further studies focusing on the effects of an Epsin triple knockout system on synaptic vesicle endocytosis would be of significant interest.

## Methods

### Cell culture and immunofluorescence

Hippocampal CA3-CA1 regions were dissected from 0–1 day-old Sprague Dawley rats, dissociated, and plated onto poly-ornithine-coated glass for 14–21 days as described previously^5^. Animal treatment was carried out in accordance with the Animal Care and Use Guidelines by Kyung Hee University. All experiments were approved by the Animal Care Committee of the Kyung Hee University. For immunofluorescence analysis of endogenous proteins, neurons were fixed 14–18 days *in vitro* (DIV) after plating. For immunofluorescence analysis of exogenous proteins, neurons were transfected with constructs (vG-pH and Epsin1-mKate2) 7–8 days after plating and fixed after 14–18 days *in vitro* (DIV) (6–10 days after transfection). Neurons were fixed with 4% paraformaldehyde, permeabilized with 0.2% Triton X-100, blocked with 5% BSA, and subsequently incubated with the appropriate primary antibodies [anti-GFP (Life Technologies), anti-vGlut1 (NeuroMab) or anti-Epsin1 (Santa Cruz)]. Alexa 488 or Alexa 546- conjugated secondary antibodies (Invitrogen) were applied to primary antibody-incubated samples with different color combinations, as needed.

### Optical setup and imaging

For immunofluorescence, images of fixed cells were acquired using a Leica DMRBE microscope through a PL APO 63x (1.32 NA) or PL Fluor 40x (1.0 N.A.) Leica objective with a CoolSNAP HQ camera (Photometrics) driven by MetaMorph software. For presynaptic terminal live imaging to examine synaptic vesicle endocytosis, constructs (vG-pH with/without shRNA-Epsin1-KD1 or shRNA-Epsin1-KD2, please see the ‘Plasmids’ section in ‘Methods’) were transfected 8 days after plating. Experiments were carried out at 14–21 DIV after plating. Coverslips were mounted in a stimulation chamber with laminar-flow perfusion on the stage of a custom-built laser-illuminated epifluorescence microscope. Live images were acquired with an Andor iXon Ultra 897 (Model #DU-897U-CS0-#BV) back-illuminated EM CCD camera. A diode-pumped OBIS 488 laser (Coherent) shuttering by synchronizing the TTL on/off signal from the EMCCD camera during acquisition was utilized as a light source. Fluorescence excitation/emission and collection were achieved using a 40x (1.3 NA) Fluar Zeiss objective lens with 500–550 nm emission and 498 nm dichroic filters (Chroma) for pHluorin. Action potentials were evoked by passing a 1 ms current pulse through platinum-iridium electrodes from an isolated current stimulator (World Precision Instruments). Neurons were perfused in saline-based buffer containing (in mM) 119 NaCl, 2.5 KCl, 2 CaCl_2_, 2 MgCl_2_, 25 HEPES, 30 glucose, 10 μM 6-cyano-7nitroquinoxaline-2,3-dione (CNQX), and 50 μM D,L-2-amino-5-phosphonovaleric acid (AP5) (adjusted to pH 7.4). All experiments were performed at 30 °C. However to examine the possible effect of temperature on synaptic vesicle endocytosis in our system, we repeated some of the endocytosis assays 25 °C and 35 °C (Supplement Figs 2 and 5). All chemicals were purchased from Sigma, unless otherwise specified. For NH_4_Cl solution application, 50 mM NaCl (pH 7.4) was substituted with 50 mM NH_4_Cl. Images for vG-pH and vG-pH with shRNA-Epsin1 KD-transfected neurons were stimulated for 2.5, 5, 10, and 30 s at 10 Hz, respectively. NH_4_Cl was applied to measure the size of the total synaptic vesicle pool. Images were acquired at 2 Hz with 50 ms exposure. To examine the rate of synaptic vesicle exocytosis, we applied bafilomycin A1 (100 μM, Calbiochem) for 30 s before we began the imaging^37^.

### Image analysis

Image J was utilized for all image analyses (http://rsb.info.nih.gov/ij). Distribution of Epsin1 at synaptic boutons was measured using Image J by selecting vGlut1-positive puncta. For pHluorin analysis, we followed previously reported methods^23^ with minor modifications. vG-pH-positive boutons were selected as the region of interest (oval, diameter: 8 pixels). Images were assessed using Image J with the plugin Time-series analyzer. Fluorescent traces were analyzed with Origin Pro 8.0. Exponential fitting was performed using a single exponential function. For statistical analysis, either one-way ANOVA (over three samples) or student t-test (two samples) was applied. Data are presented as means ± S.E.M (standard error of mean).

### Plasmids and transfection

Rat *Epsin1* cDNA was obtained from Addgene (ID#21066). Oligonucleotides for plasmid construction are listed in Supplementary Table 1. For constructing Epsin1-mKate2 or HA-Epsin1, Epsin1 was amplified via PCR using the appropriate primers (supplementary Table1) and subcloned in-frame into the pmKate2-N vector (Evrogen). For shRNA-Epsin1, oligonucleotides containing Epsin1-targeting sequences (supplementary Table 2) were synthesized, annealed, and the resulting construct ligated into pSuper vector using *Bgl*II and *Hind*III or *Xho*I restriction sites, according to the manufacturer’s instructions. shRNA-resistant Epsin1 was generated using QuikChange site-directed mutagenesis (Stratagene) with HA-Epsin1 as the template (supplementary Table1). The nucleotide sequence corresponding to the target sequence of shRNA-Epsin1 was mutated, leaving the amino acid sequence unchanged. Domain deletion mutants of Epsin1 were amplified via PCR using the appropriate primers (supplementary Table 1) and shRNA-resistant Epsin1 as template, and subcloned in-frame into pEGFP-N1 in which EGFP cDNA was replaced with mCherry. vG-pH was described previously^39^, and VAMP2-pH and synaptophysin-pH from Tim Ryan (Weill Cornell Medical College).

The Ca^2+^ phosphate precipitation method was utilized for transfection into hippocampal neurons as described previously^21^. Plasmids were incubated with Ca^2+^ (2 mM), 2x HeBS (273 NaCl, 9.5 KCl, 1.4 Na_2_HPO_4_.7H_2_O, 15 D-glucose, 42 HEPES (in mM), pH 7.10, and the mixture subsequently applied to 8 DIV hippocampal neurons.

## Additional Information

**How to cite this article**: Kyung, J. W. *et al*. Epsin1 modulates synaptic vesicle retrieval capacity at CNS synapses. *Sci. Rep.* **6**, 31997; doi: 10.1038/srep31997 (2016).

## Supplementary Material

Supplementary Information

## References

[b1] Zhang Q., Li Y. & Tsien R. W. The dynamic control of kiss-and-run and vesicular reuse probed with single nanoparticles. Science 323, 1448–53 (2009). 19213879 10.1126/science.1167373 PMC2696197

[b2] Clayton E. L. & Cousin M. A. The molecular physiology of activity-dependent bulk endocytosis of synaptic vesicles. J Neurochem 111, 901–14 (2009). 19765184 10.1111/j.1471-4159.2009.06384.x PMC2871311

[b3] Watanabe S. . Ultrafast endocytosis at mouse hippocampal synapses. Nature 504, 242–7 (2013). 24305055 10.1038/nature12809 PMC3957339

[b4] Granseth B., Odermatt, , Royle S. J. & Lagnado L. Clathrin-mediated endocytosis is the dominant mechanism of vesicle retrieval at hippocampal synapses. Neuron 51, 773–86 (2006). 16982422 10.1016/j.neuron.2006.08.029

[b5] Kim S. H. & Ryan T. A. Synaptic vesicle recycling at CNS snapses without AP-2. J Neurosci 29, 3865–74 (2009). 19321783 10.1523/JNEUROSCI.5639-08.2009 PMC2713063

[b6] Cremona O. . Essential role of phosphoinositide metabolism in synaptic vesicle recycling. Cell 99, 179–88 (1999). 10535736 10.1016/s0092-8674(00)81649-9

[b7] Mani M. . The dual phosphatase activity of synaptojanin1 is required for both efficient synaptic vesicle endocytosis and reavailability at nerve terminals. Neuron 56, 1004–18 (2007). 18093523 10.1016/j.neuron.2007.10.032 PMC3653591

[b8] Milosevic I. . Recruitment of endophilin to clathrin-coated pit necks is required for efficient vesicle uncoating after fission. Neuron 72, 587–601 (2011). 22099461 10.1016/j.neuron.2011.08.029 PMC3258500

[b9] Chen H. . Epsin is an EH-domain-binding protein implicated in clathrin-mediated endocytosis. Nature 394, 793–7 (1998). 9723620 10.1038/29555

[b10] Itoh T. . Role of the ENTH domain in phosphatidylinositol-4,5-bisphosphate binding and endocytosis. Science 291, 1047–51 (2001). 11161217 10.1126/science.291.5506.1047

[b11] Polo S. . A single motif responsible for ubiquitin recognition and monoubiquitination in endocytic proteins. Nature 416, 451–5 (2002). 11919637 10.1038/416451a

[b12] Oldham, , Mohney R. P., Miller S. L., Hanes, & O’Bryan J. P. The ubiquitin-interacting motifs target the endocytic adaptor protein epsin for ubiquitination. Curr Biol 12, 1112–6 (2002). 12121618 10.1016/s0960-9822(02)00900-4

[b13] Wendland B., Steece K. E. & Emr S. D. Yeast epsins contain an essential N-terminal ENTH domain, bind clathrin and are required for endocytosis. Embo J 18, 4383–93 (1999). 10449404 10.1093/emboj/18.16.4383 PMC1171513

[b14] Ford M. G. . Curvature of clathrin-coated pits driven by epsin. Nature 419, 361–6 (2002). 12353027 10.1038/nature01020

[b15] Itoh T. & De Camilli P. BAR, F-BAR (EFC) and ENTH/ANTH domains in the regulation of membrane-cytosol interfaces and membrane curvature. Biochim Biophys Acta 1761, 897–912 (2006). 16938488 10.1016/j.bbalip.2006.06.015

[b16] Bao H., Reist N. E. & Zhang B. The Drosophila epsin 1 is required for ubiquitin-dependent synaptic growth and function but not for synaptic vesicle recycling. Traffic 9, 2190–205 (2008). 18796008 10.1111/j.1600-0854.2008.00832.x

[b17] Chen C. & Zhuang X. Epsin 1 is a cargo-specific adaptor for the clathrin-mediated endocytosis of the influenza virus. Proc Natl Acad Sci USA 105, 11790–5 (2008). 18689690 10.1073/pnas.0803711105 PMC2504482

[b18] Shen, . Phagocytic receptor signaling regulates clathrin and epsin-mediated cytoskeletal remodeling during apoptotic cell engulfment in C. elegans. Development 140, 3230–43 (2013). 23861060 10.1242/dev.093732 PMC3931732

[b19] Kang Y. L. . Caenorhabditis elegans reveals a FxNPxY-independent low-density lipoprotein receptor internalization mechanism mediated by epsin1. Mol Biol Cell 24, 308–18 (2012). 23242996 10.1091/mbc.E12-02-0163 PMC3564534

[b20] Jakobsson J. . Role of epsin 1 in synaptic vesicle endocytosis. Proc Natl Acad Sci USA 105, 6445–50 (2008). 18430801 10.1073/pnas.0710267105 PMC2359800

[b21] Kim S. H. & Ryan T. A. CDK5 serves as a major control point in neurotransmitter release. Neuron 67, 797–809 (2010). 20826311 10.1016/j.neuron.2010.08.003 PMC2939042

[b22] Kim S. H. & Ryan T. A. A distributed set of interactions controls mu2 functionality in the role of AP-2 as a sorting adaptor in synaptic vesicle endocytosis. J Biol Chem 284, 32803–12 (2009). 19762466 10.1074/jbc.M109.039149 PMC2781697

[b23] Kim S. H. & Ryan T. A. Balance of Calcineurin Aalpha and CDK5 Activities Sets Release Probability at Nerve Terminals. J Neurosci 33, 8937–50 (2013). 23699505 10.1523/JNEUROSCI.4288-12.2013 PMC3808255

[b24] Voglmaier S. M. . Distinct endocytic pathways control the rate and extent of synaptic vesicle protein recycling. Neuron 51, 71–84 (2006). 16815333 10.1016/j.neuron.2006.05.027

[b25] Balaji J. & Ryan T. A. Single-vesicle imaging reveals that synaptic vesicle exocytosis and endocytosis are coupled by a single stochastic mode. Proc Natl Acad Sci USA 104, 20576–81 (2007). 18077369 10.1073/pnas.0707574105 PMC2154473

[b26] Dittman J. S. & Kaplan J. M. Factors regulating the abundance and localization of synaptobrevin in the plasma membrane. Proc Natl Acad Sci USA 103, 11399–404 (2006). 16844789 10.1073/pnas.0600784103 PMC1544097

[b27] Sankaranarayanan S. & Ryan T. A. Calcium accelerates endocytosis of vSNAREs at hippocampal synapses. Nat Neurosci 4, 129–36 (2001). 11175872 10.1038/83949

[b28] Schweizer F. E. & Ryan T. A. The synaptic vesicle: cycle of exocytosis and endocytosis. Curr Opin Neurobiol 16, 298–304 (2006). 16707259 10.1016/j.conb.2006.05.006

[b29] Messa M. . Epsin deficiency impairs endocytosis by stalling the actin-dependent invagination of endocytic clathrin-coated pits. Elife 3, e03311 (2014). 25122462 10.7554/eLife.03311 PMC4161027

[b30] Balaji J., Armbruster M. & Ryan T. A. Calcium control of endocytic capacity at a CNS synapse. J Neurosci 28, 6742–9 (2008). 18579748 10.1523/JNEUROSCI.1082-08.2008 PMC2671224

[b31] Takamori S. . Molecular anatomy of a trafficking organelle. Cell 127, 831–46 (2006). 17110340 10.1016/j.cell.2006.10.030

[b32] Tessneer K. L. . Endocytic adaptor protein epsin is elevated in prostate cancer and required for cancer progression. ISRN Oncol 2013, 420597 (2013). 23691361 10.1155/2013/420597 PMC3649151

[b33] Tessneer, . Epsin Family of Endocytic Adaptor Proteins as Oncogenic Regulators of Cancer Progression. J Can Res Updates 2, 144–150 (2014). 10.6000/1929-2279.2013.02.03.2 PMC3911794 24501612

[b34] Pasula S. . Endothelial epsin deficiency decreases tumor growth by enhancing VEGF signaling. J Clin Invest 122, 4424–38 (2012). 23187125 10.1172/JCI64537 PMC3533553

[b35] Tian X., Hansen D., Schedl T. & Skeath J. B. Epsin potentiates Notch pathway activity in Drosophila and C. elegans. Development 131, 5807–15 (2004). 15539484 10.1242/dev.01459

[b36] Overstreet E., Chen X., Wendland B. & Fischer J. A. Either part of a Drosophila epsin protein, divided after the ENTH domain, functions in endocytosis of delta in the developing eye. Curr Biol 13, 854–60 (2003). 12747835 10.1016/s0960-9822(03)00326-9

[b37] Kim, & Ryan T. A. Synaptic vesicle recycling at CNS synapses without AP-2. J Neurosci 29, 3865–74 (2009). 19321783 10.1523/JNEUROSCI.5639-08.2009 PMC2713063

[b38] Chen H., Polo, , Di Fiore P. P. & De Camilli P. V. Rapid Ca2+-dependent decrease of protein ubiquitination at synapses. Proc Natl Acad Sci USA 100, 14908–13 (2003). 14657369 10.1073/pnas.2136625100 PMC299851

[b39] Cottrell, . Calcineurin Agamma is a Functional Phosphatase That Modulates Synaptic Vesicle Endocytosis. J Biol Chem 291, 1948–56 (2016). 26627835 10.1074/jbc.M115.705319 PMC4722470

