# Supplementary material for: Epsin1 modulates synaptic vesicle retrieval capacity at CNS synapses
Source: Sci Rep. 2016 Aug 25;6:31997. doi: 10.1038/srep31997 (PMC4997357; doi:10.1038/srep31997)
Supplement: Supplementary Information [file srep31997-s1.pdf]

Supplementary Information

Epsin1 modulates synaptic vesicle retrieval capacity at CNS synapses

Jae Won Kyung[1], Jae Ryul Bae[1], Dae-Hwan Kim[4], Woo Keun Song[5], Sung Hyun Kim[2,3 *]

[1]Department of Biomedical Science, Graduate School, Kyung Hee University, [2]Department of Physiology, Kyung Hee University, School of Medicine, [3]Neurodegeneration Control Research Center, Seoul, 01726, South Korea. [4]Institute of Pharmaceutical Science and Technology, Hanyang University, Ansan, 15588, South Korea. [5]School of Life Science, Bio Imaging and Cell Dynamics Research Center, Gwangju Institute of Science and Technology, Gwangju 61005, South Korea.

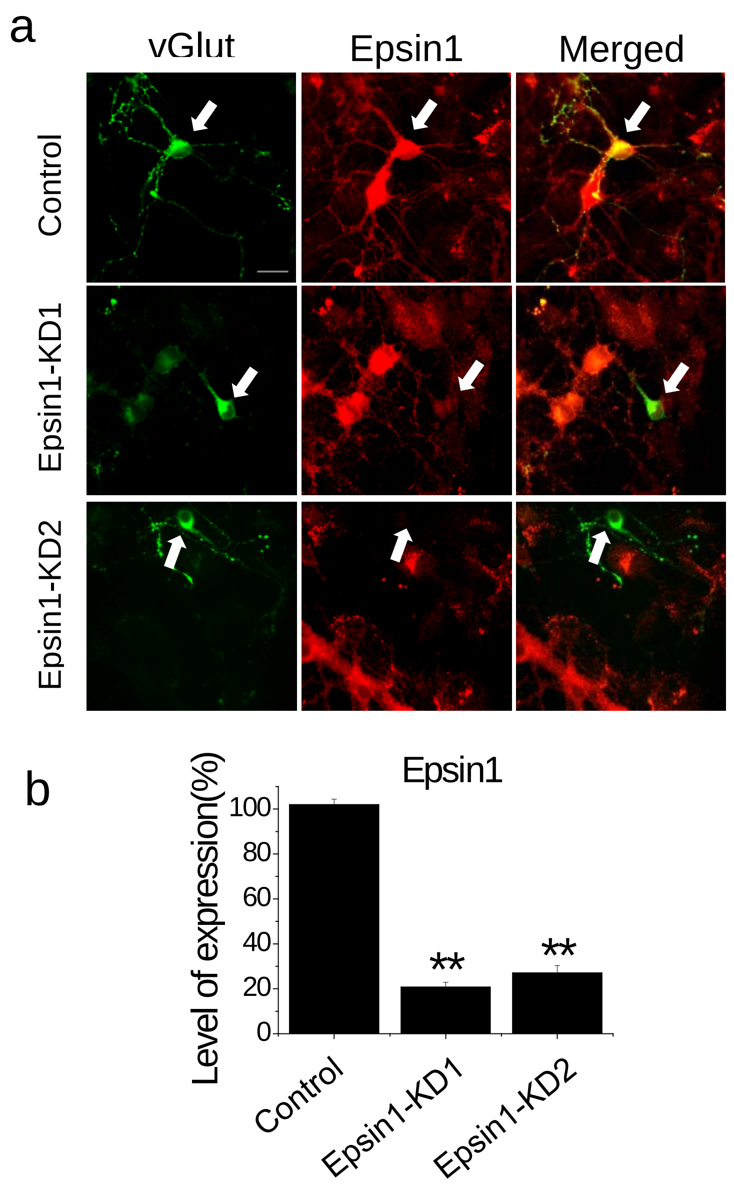

Supplementary Figure 1. shRNA targeting Epsin1 efficiently downregulates expression of Epsin1 in primary cultured hippocampal neurons

(a) Representative images of hippocampal neurons simultaneously stained with anti-GFP (green) and anti-Epsin1 (red). Neurons were transfected with vG-pH with or without shRNA targeting Epsin1 (Epsin1-KD1 or Epsin1-KD2) 8 days after plating. After 6-10 days, neurons were fixed and subsequently stained with anti-GFP and anti-Epsin1 antibodies. The arrow bar indicates transfected neurons under each experimental condition. Scale bar, 50 μm. (b) Mean values of Epsin1 expression in control and Epsin1-KD neurons. Cells transfected with shRNA targeting Epsin1 (Epsin1-KD1 or Epsin1-KD2) were significantly depleted of Epsin1, compared to control cells. $[Epsin1]_{con}$ =102.00 ± 2.38% (n= 11), $[Epsin1]_{Epsin1\text{-}KD1}$ = 20.80 ± 2.08% (n=8), $[Epsin1]_{Epsin1\text{-}KD2}$ = 27.15 ± 3.17% (n=13). ***p*<0.01 one-way ANOVA

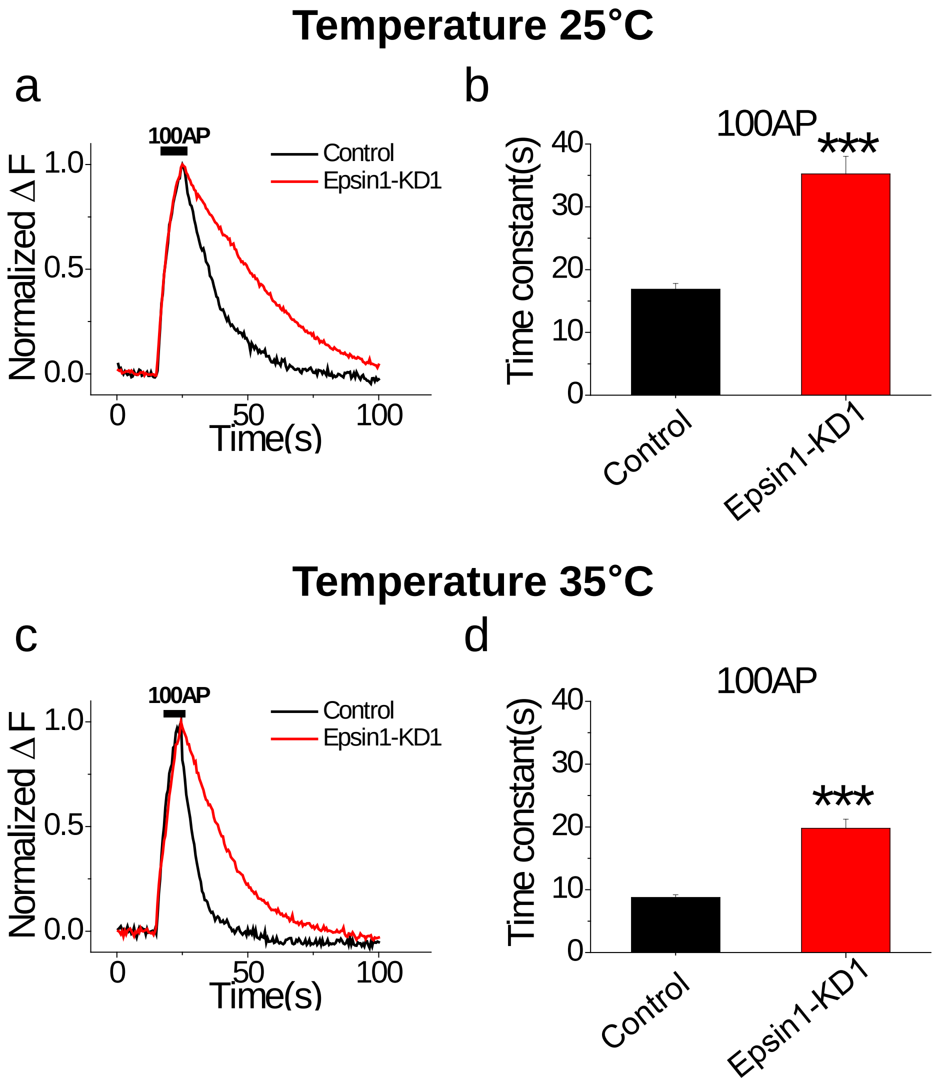

Supplementary Figure 2. Synaptic vesicle endocytosis is altered in Epsin1 KD neurons stimulated under various temperature conditions

(a and c) Representative traces of endocytosis from control (black) and Epsin1-KD1 (red) neurons at 25°C (a) and 35°C (c). Neurons transfected with vG-pH with/without Epsin1-KD1 were stimulated at 10Hz for 10s (100AP) at 25°C and 35°C. (b and d) Mean time constants of endocytosis obtained from control and Epsin1-KD1 neurons at 25°C (b) and 35°C (d). (25°C: $\tau_{endo\ con}$ =16.87 s ± 0.92 s, n= 8, $\tau_{endo\ Epsin1\ KD1}$ = 35.24 s ± 2.80, n= 10; 35°C: $\tau_{endo\ con}$ =8.76 s ± 0.44 s, n= 8, $\tau_{endo\ Epsin1\ KD1}$ = 19.77 s ± 1.48, n= 10). ****p*<0.001, student t-test.

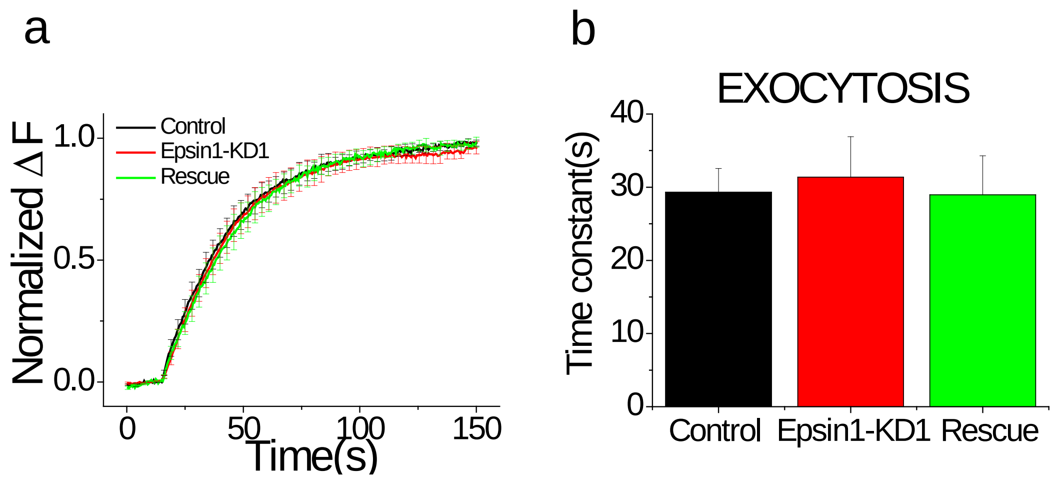

Supplementary Figure 3. Rate of exocytosis is not defected in Epsin1 depleted neurons.

(a) Ensemble average traces of exocytosis of vG-pH in control, Epsin1-KD1, and rescue neurons. Neurons were stimulated with 1200 AP at 10Hz in the presence of bafilomycin A1 to acquire pure exocytosis of vesicle. (b) Mean values of rate of exocytosis in control, Epsin1-KD1, and rescue neurons. The rate of exocytosis was determined using a single exponential fit. Time constants for exocytosis in control, Epsin1-KD1, and rescue neurons are not significantly different. $\tau_{\text{exo con}}$ = 29.32 ± 3.22 s (n=9), $\tau_{\text{exo Epsin1-KD1}}$ = 31.38 ± 5.50 s (n=9), $\tau_{\text{exo res}}$ = 28.96 ± 5.32 s (n=7).

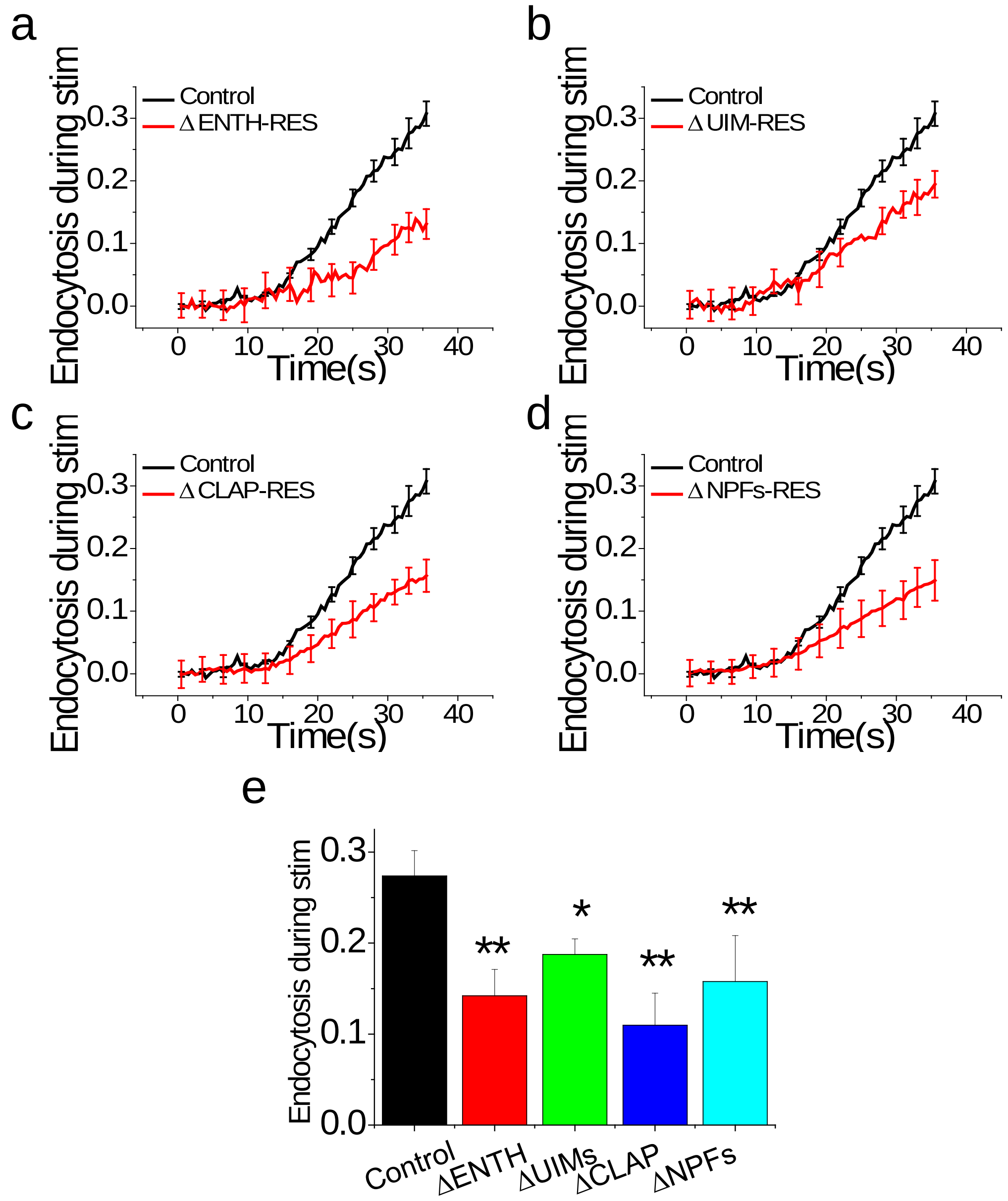

Supplementary Figure 4. Domain deletion mutants have a defect in endocytosis during neural activities

(a-d) average amount of endocytosis during neural activities as function of time in control (black) and deletion domain mutant-replaced (red) neurons. ΔENTH-res (a), ΔUIMs-res (b), ΔCLAP-res (c), and ΔNPFs-res (d), respectively. (e) Mean values of the amount of endocytosis during stimulation at 30s. Each deletion mutant replaced neurons reveal significant decrease of endocytosis during stimulation. Endocytosis during stimulation was analyzed as $\Delta F_{300Baf+}$ - $\Delta F_{300Baf-}$. $[endo]_{Con}$ = 27.3 ± 2.7 % (n=9), $[endo]_{Epsin1-\Delta ENTH-res}$ = 14.20 ± 0.2 % (n=9), $[endo]_{Epsin1-\Delta UIMs-res}$ = 18.74 ± 1.7 % (n=9), $[endo]_{Epsin1-\Delta CLAP-res}$ = 10.98 ± 3.5 % (n=9), $[endo]_{Epsin1-\Delta NPFs-res}$ = 15.77 ± 5.0 % (n=9), *p<0.05, **p<0.01, one-way ANOVA

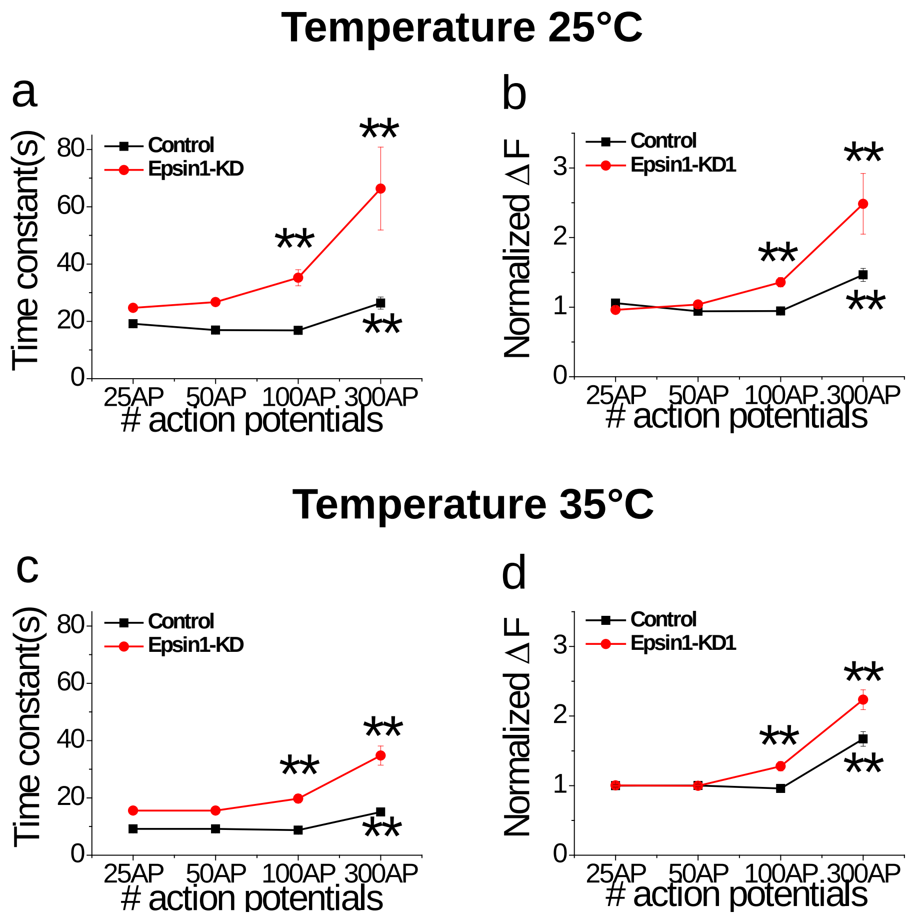

Supplementary Figure 5. The endocytic capacity is altered in Epsin1-KD1 neurons subjected to various stimulations at different temperatures

The kinetics of post-stimulus endocytosis were measured at 25°C (a and b) and 35°C (c and d) in neurons subjected to a range of stimuli (25, 50, 100, and 300 AP). (a and c) Mean values of the endocytic time constants obtained at 25°C (a) and 35°C (c) from control and Epsin1-KD1 neurons stimulated with 25, 50, 100 and 300 AP. The time constant of Epsin1-KD1 neurons is altered beginning at 100AP, while that in control neurons begins at 300 AP. 25°C : $\tau_{endo\ con\ 25AP}$ = 19.17 ± 1.22 s, $\tau_{endo\ con\ 50AP}$= 16.96 ± 0.99 s, $\tau_{endo\ con\ 100AP}$= 16.97 ± 0.92 s, $\tau_{endo\ con\ 300AP}$= 26.36 ± 2.11 s, n=8 ; $\tau_{endo\ Epsin1\text{-}KD1\ 25AP}$= 24.71 ± 1.08 s, $\tau_{endo\ Epsin1\text{-}KD1\ 50AP}$= 26.72 ± 1.24 s, $\tau_{endo\ Epsin1\text{-}KD1\ 100AP}$= 35.24 ± 2.80 s, $\tau_{endo\ Epsin1\text{-}KD1\ 300AP}$= 66.34 ± 14.47 s, n=10. 35°C: $\tau_{endo\ con\ 25AP}$ = 9.20 ± 0.58 s, $\tau_{endo\ con\ 50AP}$= 9.18 ± 0.46 s, $\tau_{endo\ con\ 100AP}$= 8.76 ± 0.44 s, $\tau_{endo\ con\ 300AP}$= 15.07 ± 0.62 s, n=8 ; $\tau_{endo\ Epsin1\text{-}KD1\ 25AP}$= 15.59 ± 0.98 s, $\tau_{endo\ Epsin1\text{-}KD1\ 50AP}$= 15.58 ± 1.07 s, $\tau_{endo\ Epsin1\text{-}KD1\ 100AP}$= 19.76 ± 1.46 s, $\tau_{endo\ Epsin1\text{-}KD1\ 300AP}$= 34.78 ± 3.34 s, n=10. (b and d) Time constants obtained from control and Epsin1-KD1 neurons under exposure to various stimuli, normalized to the average value obtained at 25-50AP under each condition. Overlay of the normalized time constants obtained under various stimulation conditions in control (black) and Epsin1-KD1 (red) neurons. The endocytic capacity is significantly decreased in Epsin1-KD1 neurons. ***p*<0.01, One-way ANOVA.

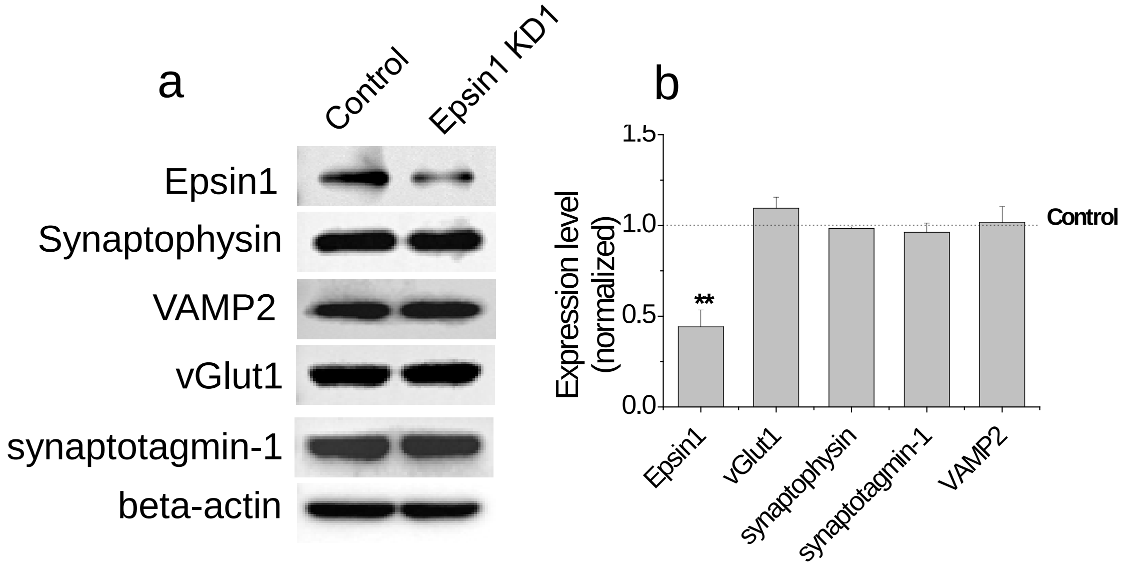

Supplementary Figure 6. The expression levels of SV cargo proteins are not altered in Epsin1 KD1 neurons

(a) Representative immunoblot showing synaptic cargo proteins in control and Epsin1 KD neurons. Neurons transfected with/without Epsin1 KD1 were lysed, subjected to SDS-PAGE, transferred to PVDF membranes, and blotted with antibodies against Epsin1 and the synaptic cargo proteins, synaptophysin, VAMP2, vGlut1, synaptotagmin-1, and beta-actin. (b) Band intensities were quantified and compared using the Image J software. ***p*<0.01, student t-test.

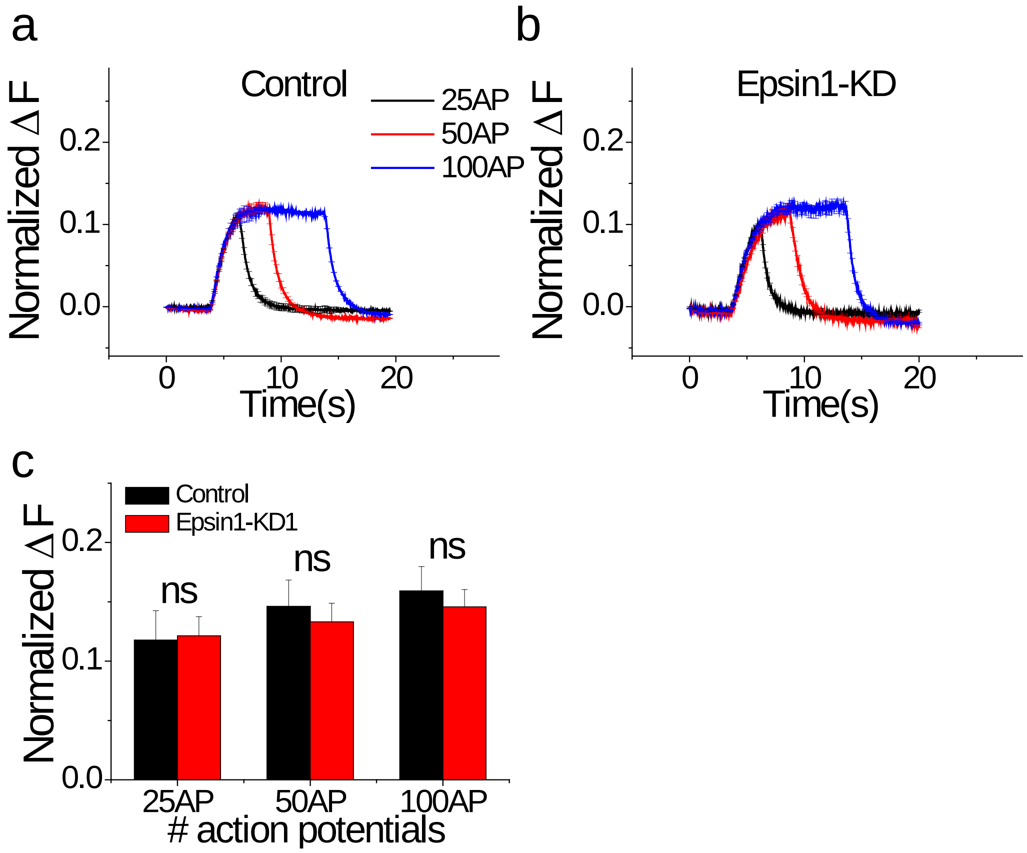

Supplementary Figure 7. Activity-dependent Ca^{2+} influx at nerve terminal is not different in between control and Epsin1-KD1 neurons.

(a-b) Ensemble average traces of synaptophysin-GCaMP6f (physin-GC6f) response to various stimuli (25, 50, 100AP) in control (a) and Epsin1-KD1 (b) neurons. Neurons transfected with physin-GC6f with/without shRNA-targeting Epsin1 were stimulated with various condition (25, 50, 100AP). (c) Mean value of peak point of physin-GC6f response to each condition in control and Epsin1-KD1 neurons. These are not significantly different. $[Ca^{2+}]_{con\ 25AP}$ =0.11 ± 0.02, $[Ca^{2+}]_{con\ 50AP}$ =0.14 ± 0.02, $[Ca^{2+}]_{con\ 100AP}$ =0.15 ± 0.02, (n=9) ; $[Ca^{2+}]_{Epsin1\text{-}KD1\ 25AP}$ =0.12 ± 0.01 , $[Ca^{2+}]_{Epsin1\text{-}KD1\ 50AP}$ =0.13 ± 0.01, $[Ca^{2+}]_{Epsin1\text{-}KD1\ 100AP}$ =0.14 ± 0.01, (n=10). ns = non-significant

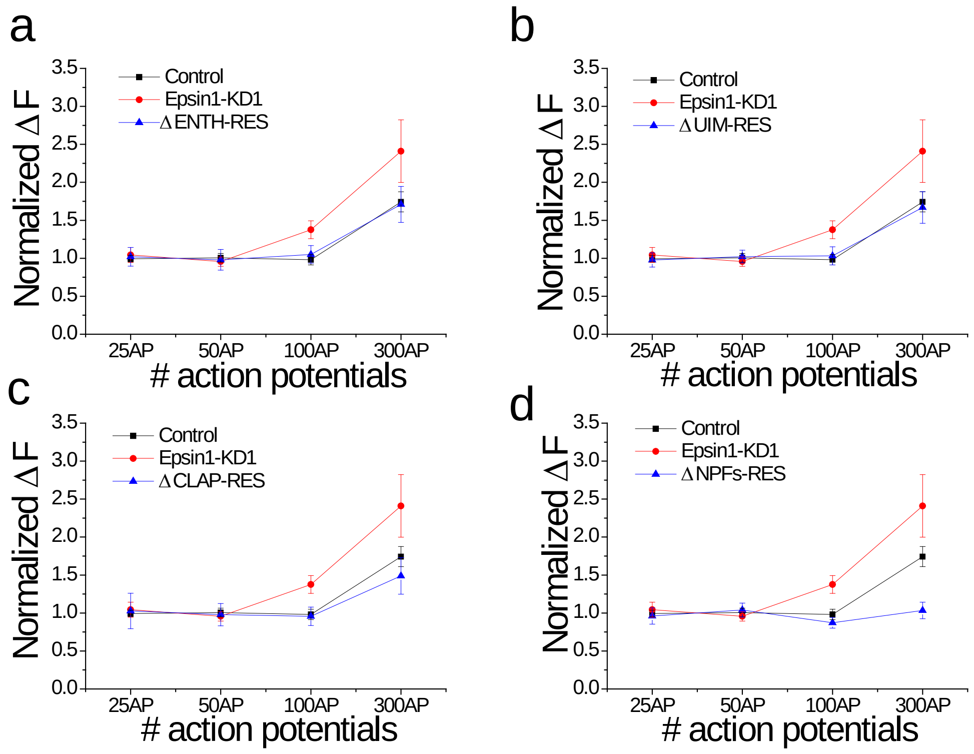

Supplementary Figure 8. Endocytic capacity is not changed by replacing deletion mutant of Epsin1 in Epsin1-KD1 neurons.

(a-d) Normalized time constants of poststimulus endocytosis under various stimuli in Epsin1-KD neurons incorporating individual Epsin1 domain deletion mutants. (a) ΔENTH, (b) ΔUIMs, (c) ΔCLAP, (d) ΔNPFs, respectively. Neurons transfected with vG-pH, shRNA1-Epsin1, and each deletion mutants (ΔENTH, ΔUIMs, ΔCLAP, or ΔNPFs) were stimulated various conditions (25, 50, 100, 300 AP at 10Hz). Results were normalized by mean value of time constant of 25 and 50AP in each condition.

Supplementary methods

Imaging and analysis of Ca^{2+} influx at presynaptic terminals

For live imaging for synaptic Ca^{2+} entry, neurons were transfected with synaptophysin-GCaMP6f with or without shRNA-Epsin1 8 days after plating. Experiments were carried out 14-21 DIV after plating. Coverslips were mounted in a stimulation chamber with laminar flow perfusion on the stage of a custom-built laser-illuminated epi-fluorescence microscope. Neurons were stimulated under various conditions (25, 50, 100, 300 AP at 10Hz) with 2 mM external Ca^{2+}. Imaging of Ca^{2+} influx was recorded at 100 Hz with 9.73 ms exposure in a frame transfer mode. Ionomycin, 200 μM (Alomone Labs), was applied to record maximum fluorescence of Physin-GC6f at the end of each experiment.

For GCaMP6f analysis, we followed the previously described method of Kim and Ryan, (2013), with minor modifications. Images were analyzed using Image J with the plugin Time-series analyzer. Fluorescent traces were analyzed with Origin Pro 8.0. For Ca^{2+} entry, physin-GC6f-positive boutons were used to mark ROI. Peak amplitudes of each response at various conditions were obtained. Peak values are normalized to that of ionomycin fluorescence.

Immunoblot analysis

Neurons were plated to a poly-ornithine-coated 12-well dish and incubated for ~14 days. On day 8th after plating, neurons were transfected with cytosolic GFP (pEGFP-N1) with/without shRNA-targeting Epsin1. At 6 days post-transfection (14 DIV), the cells were lysed with lysis buffer containing 10 mM Tris (pH 7.4), 1% SDS, 10 mM NaF, 1 mM PMSF, and protease inhibitors (Complete Mini; Roche, Germany). Total proteins were quantified using the bicinchoninic acid (BCA) assay (Thermo Scientific) and equal amounts of protein (20 μg) from control and Epsin1 KD1 cells were subjected to SDS-PAGE and subsequently transferred to PVDF membranes. The membranes were incubated for 1 h with blocking solution containing 5% non-fat dry milk, and then with the appropriate primary antibodies. The utilized primary antibodies were as follows: anti-Epsin1 from Santa Cruz; anti-synaptophysin, anti-VAMP2, and anti-synaptotagmin-1 from Synaptic System; anti-vGlut-1 from NeuroMab; and anti-beta actin from Abcam. Band intensities were quantified using the Image J software and normalized with respect to that of beta-actin. The experiments were repeated at least three times.

Supplementary tables

Table1. Primers for Epsin1 deletion mutant

Name	Forward Primer	Reverse Primer
Epin1-mKate2	GATGATGGATTCATGTCGACATCATCG	GATGATACCGGTAGTAGGAGGAAGGGGT
HA-Epsin1	AATAATGAATTCATGTCGACATCATCG	ATAACTCTCGAGTTATAGGAGGAAGGG
shRNA-resistant-Epsin1	GTTGTGAAGACCTCGGCTAGTTCCATAAGAG	CTCTTATGGAACTAGCCGAGGTCTTCACAAC
Epsin1-ΔENTH	GATGATACCGGTCATAGGAGGAAGGGGT	GATGATGAATTCATGGCCCACGCGCTCAAG
Epsin1-ΔUIM	GGAATGGGTTCTTGCCCCCAGTCTC	GGAGGAGCGCGACCCTTGGGGGGGC
Epsin1-ΔCLAP	GGAATGGGTTCTTGCCCCCAGTCTC	TGGGGGCAAGAACCCATTCCTTCCA
Epsin1-ΔNPFs	GATGATACCGGTCAGGAAGCCTTAACTC	GATGATGAATTCATGTCGACATCA TCG

Table2. Target sequence for shRNAs-Epsin1

Name	Target sequence
shRNA1-Epsin1(Epsin1-KD1)	GGATCTTGCTGACGTCTTC
shRNA2-Epsin1(Epsin1-KD2)	CAACGACCATGGCAAGAATT
